# Efficacy of Carotenoid-Loaded Gelatin Nanoparticles in Reducing Plasma Cytokines and Adipocyte Hypertrophy in Wistar Rats

**DOI:** 10.3390/ijms241310657

**Published:** 2023-06-26

**Authors:** Jaluza Luana C. de Queiroz, Isaiane Medeiros, Mayara S. R. Lima, Fabiana Maria C. de Carvalho, Christina S. Camillo, Pedro Paulo de A. Santos, Gerlane C. B. Guerra, Valéria C. da Silva, Helena T. Schroeder, Mauricio Krause, Ana Heloneida de A. Morais, Thaís S. Passos

**Affiliations:** 1 Biochemistry and Molecular Biology Postgraduate Program, Biosciences Center, Federal University of Rio Grande do Norte, Natal 59078-970, Brazil; 2 Nutrition Course, Potiguar University, Natal 59056-000, Brazil; 3 Postgraduate Program in Structural and Functional Biology, Biosciences Center, Federal University of Rio Grande do Norte, Natal 59078-970, Brazil; 4 Development and Technological Innovation in Medicines Postgraduate Program, Center for Health Sciences, Federal University of Rio Grande do Norte, Natal 59078-970, Brazil; 5 Laboratory of Inflammation, Metabolism and Exercise Research (LAPIMEX) and Laboratory of Cellular Physiology, Department of Physiology, Institute of Basic Health Sciences, Federal University of Rio Grande do Sul, Porto Alegre 90050-170, Brazil; 6 Nutrition Postgraduate Program, Center for Health Sciences, Federal University of Rio Grande do Norte, Natal 59078-970, Brazil

**Keywords:** *Cucumis melo* L., nanoparticles, anti-inflammatory agents, adiposity

## Abstract

The present study investigated the effect of gelatin-based nanoparticles (EPG) loaded with a carotenoid-rich crude extract (CE) on systemic and adipose tissue inflammatory response in a model with inflammation induced by a high glycemic index and high glycemic load diet (HGLI). Nanoparticles synthesized were characterized by different physical and chemical methods. The in vivo investigation evaluated Wistar rats (n = 20, 11 days, adult male with 21 weeks) subdivided into untreated (HGLI diet), conventional treatment (nutritionally adequate diet), treatment 1 (HGLI + crude extract (12.5 mg/kg)), and treatment 2 (HGLI + EPG (50 mg/kg)) groups. Dietary intake, caloric intake and efficiency, weight, inflammatory cytokines tissue concentration, visceral adipose tissue (VAT) weight, histopathological analysis, and antioxidant activity in plasma and VAT were investigated. EPG showed the same physical and chemical characteristics as previous batches (95.2 nm, smooth surface, and chemical interactions between materials). The EPG-treated group was the only group promoting negative ∆dietary intake, ∆caloric efficiency, and ∆weight. In addition, it presented a significant reduction (*p* < 0.05) in IL-6 and leptin levels and a greater presence of multilocular adipocytes. The results suggest that EPG can act as a nutraceutical in adjuvant therapy for treating inflammatory diseases associated with adipose tissue accumulation.

## 1. Introduction

With the nutritional transition, there was an increase in the intake of highly palatable foods, sources of fat, and processed carbohydrates with a high glycemic index. On the other hand, a reduction in energy expenditure causes excess body fat in the population [1].

This excess body fat is characterized by increased size (hypertrophy) and the number of adipocytes (hyperplasia) responsible for fat storage and chronic inflammatory response. The latter is caused by several correlated factors, such as increased concentrations of circulating fatty acids, hypertrophied adipocyte hypoxia, and local and systemic oxidative stress [2].

Thus, the excess adipose tissue affects various biochemical events, such as increased secretion of inflammatory cytokines and leptin and decreased adiponectin concentrations in circulation, causing disorders in adipocytes, infiltration of inflammatory cells (such as macrophages), and local and systemic inflammation [3].

From this perspective, one of the classic strategies to control this serious public health problem is the recommendation of good food choices. Diets balanced and properly nutritionally adequate influence weight control, body fat, and inflammatory state [4]. Additionally, the search for adjuvant alternatives, combined with adherence to good eating habits, has been gaining ground in research related to this theme.

Thus, bioactive molecules or compounds with antioxidant action can be considered candidates, as it has a beneficial effect on preventing inflammatory state. A well-documented option in the literature is carotenoids, defined as natural pigments responsible for yellow, orange, and red observed in fruits and vegetables, being metabolites produced by plants and microorganisms [5].

Studies show that individuals with obesity and associated diseases have decreasing concentrations of carotenoids associated with a growing number of complications, thus suggesting that these molecules have a protective effect on this disease [6].

It is important to note that carotenoids act in the adipose tissue in the following ways: the adipogenesis process, in which the β-carotene is converted to β-apo-14′-carotene, which represses the activation of PPARγ, PPARα, and RXR, and acts in differentiation and limitation of lipid accumulation in adipocytes. Moreover, inducing the beige of white adipose tissue acquiring thermogenic capacity and lipid catabolism, resulting in its anti-adipogenic action and improved obesity condition [2].

Humans do not synthesize these bioactive compounds with a series of biological activities, such as anti-inflammatory activity, so their ingestion and/or supplementation is required [7].

Among the food source of these pigments is the Cantaloupe (*Cucumis melo* L. var cantalupensis) melon, which has orange pulp, indicating the presence of α-carotene and β-carotene, among other carotenoids, in its nutritional composition. However, these compounds have a lipophilic nature and a high instability to several factors related to processing, storage, and digestion in the gastrointestinal tract. They also have low bioaccessibility and bioavailability in food, which is affected by several factors (food matrix, fat, fat-soluble micronutrients, lifestyle, etc.), being necessary for a high concentration of the active compounds to promote the desired effect, limiting their applicability and justifying the use of strategies aimed at protecting these pigments. This thus improves their use and absorption by the body [8,9,10].

Therefore, a way to increase the potential for these compounds is to isolate these food matrix substances and their possible use as nutraceuticals. From this perspective, aiming to promote solubility in an aqueous matrix, increase the stability, bioaccessibility, and bioavailability of these bioactive compounds and promote the preservation and increased anti-inflammatory action, a possible strategy has been based on the nanoencapsulation of these substances [11,12]. This technique also makes it possible to reduce the number of bioactive compounds necessary to achieve the desired effect on human health, in addition to promoting the controlled release of the local target of action and providing good permeability and improvements in the distribution in the body [13].

The previous studies of the Research Group Nutrition and Bioactive Substances for Health (NutriSBioativoS) from the Federal University of Rio Grande do Norte, Brazil, have evaluated the action of the crude extract rich in carotenoids from Cantaloupe melons (*Cucumis melo* L. var cantalupensis) (CE) nanoencapsulated in porcine gelatin (EPG) by in vitro and in vivo studies. The results demonstrated the improvement in water dispersibility, the increased yellow color stability of these compounds added to yogurt [14], and the stability of in vitro antioxidant action [15]. In addition, in vivo studies showed that EPG had low toxicity and potential bioactive effect in different organs (small intestine, liver, kidneys, spleen, and stomach) [16]. It also promoted the increased concentration of retinol in the liver of animals with obesity induced by a high glycemic index diet and high glycemic load [17].

Based on this, the present study aimed to investigate the effect of the extract rich in carotenoids from Cantaloupe melons nanoencapsulated in porcine gelatin in adipose tissue and the inflammatory response of animals with chronic systemic inflammation induced by a high glycemic index diet and high glycemic load (HGLI).

## 2. Results

### 2.1. Characterization of the Crude Extract Rich in Carotenoids from Cantaloupe Melons

The line equation (y = 1.3997x + 0.0022/R^2^ = 0.9995) was obtained from the constructed calibration curve, which was used to determine the total carotenoids present in EPG. Thus, the carotenoid content equivalent to 44.88 (0.10) μg of total carotenoids/g of Cantaloupe melon pulp was obtained.

The linear equation obtained through the calibration curve using the beta-carotene standard was used to evaluate the content present in EPG. The result corresponded to 26.0 (0.00) μg of β-carotene/g melon pulp (Figure S1).

### 2.2. Characterization of Nanoparticles and Incorporation Efficiency (%)

**Figure 1:**
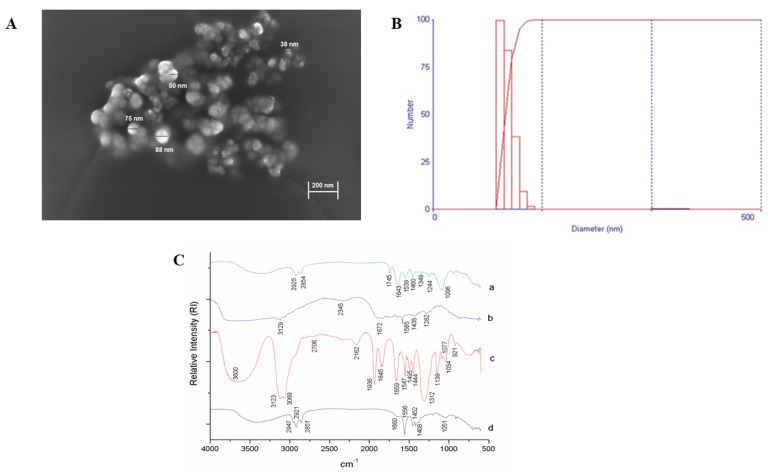
Characterization of gelatin-based nanoparticles containing carotenoid-rich extract of Cantaloupe melons obtained by the O/W emulsification technique. (**A**) Representative Scanning Electron Microscopy image. (**B**) Laser diffraction. (**C**) Fourier Transform Spectroscopy: a—EPG: crude extract rich in carotenoids from Cantaloupe melons nanoencapsulated in porcine gelatin; b—porcine gelatin; c—Tween 20; d—CE: crude extract rich in carotenoids from Cantaloupe melons. O/W: oil in water.

In the SEM image obtained for EPG, it can be observed that there was the formation of an agglomerate of spherical particles with a smooth surface, without pores, with sizes smaller than 100 nm (Figure 1A). The mean diameter and polydispersion index obtained for the EPG were, respectively, 95.2 nm (11.25) and 0.375 (0.027) (Figure 1B).

From the spectrum obtained for the crude extract of carotenoids (Figure 1C), it is possible to observe the presence of characteristic bands of hydrocarbons, such as alkenes and alkynes (1660, 1556, 1452 and 1408 cm^−1^), with stretching vibration and flexion in the C–C and C–H bonds. For Tween 20, characteristic bands were observed in the range of 3600–3200 cm^−1^, characteristics of the hydroxyl group (-OH), and this group can also be observed in the range of 1200 to 1000 cm^−1^ (1139, 1077, and 1034 cm^−1^). The range between 1936 and 921 cm^−1^ indicates the presence of C=O bonds (1845 and 1659 cm^−1^) and hydrocarbons (921 cm^−1^).

The spectrum obtained for porcine gelatin showed characteristic bands at 1672 cm^−1^ (amide I (C=O bond elongation)) and 1585 cm^−1^ (amide II, or more precisely, N–H bond) (Figure 1C). It was possible to notice that the spectrum obtained for the EPG points to the presence of new vibrational bands (1745 cm^−1^; 1349 cm^−1^), which are not visualized in the raw materials (Figure 1C), or displacement of previously observed bands in the spectra of the materials used. The incorporation efficiency obtained for the EPG was equivalent to 95 (1.66)%.

### 2.3. Preclinical Study

#### 2.3.1. Food Consumption, Caloric Intake, Caloric Efficiency, and Body Weight

Regarding the values of intake and caloric efficiency, it is possible to observe that, although there is no statistically significant difference between the groups (*p* > 0.05), the EPG-treated group showed a negative variation in dietary intake and caloric efficiency (Table 1).

Concerning body weight variation (Table 2), a balance between weight gain and loss was observed in the animals in the untreated group. On the other hand, the weight loss was observed to exceed the gain for the animals that received the conventional and the CE treatment. Furthermore, the group of animals treated with EPG was the only one that did not show weight gain over the 11 days of the experiment, for which only weight loss was observed.

#### 2.3.2. Plasma Concentrations of IL-6, TNF-α and Leptin

**Figure 2:**
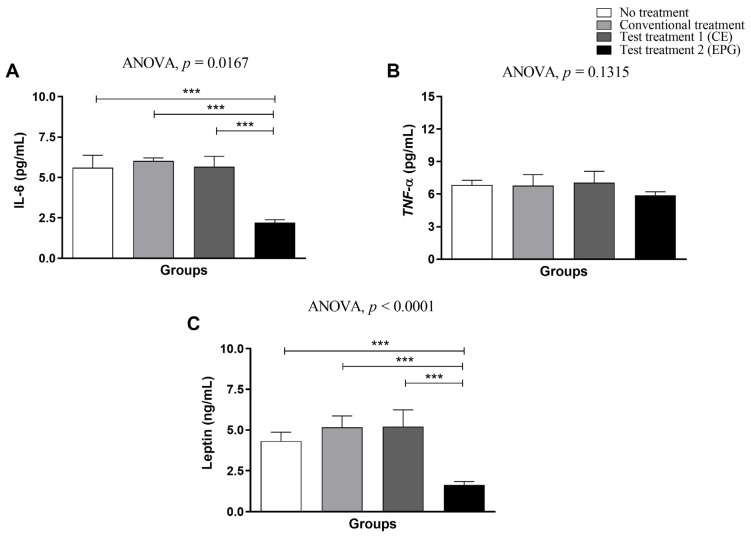
Plasma concentrations of IL-6, TNF-α, and leptin in adult male Wistar rats (31 weeks) with inflammation induced by the HGLI diet submitted to different treatments and evaluated after ten days of the experiment (11th day). (**A**) IL-6. (**B**) TNF-α. (**C**) Leptin. No treatment: HGLI diet + 1 mL of water by gavage; conventional treatment: nutritionally adequate diet (Labina^®^ feed) + 1 mL of water per gavage; test treatment 1: HGLI diet + 1 mL of CE at a concentration of 12.5 mg/kg by gavage; test treatment 2: HGLI diet + 1 mL of EPG at a concentration of 50 mg/kg by gavage. HGLI diet: mixture composed of Labina^®^, condensed milk, and sugar (1:1:0.21 *w*/*w*/*w*); HGLI: high glycemic index and high glycemic load diet; CE: crude extract rich in carotenoids from Cantaloupe melons; EPG: crude extract rich in carotenoids from Cantaloupe melons nanoencapsulated in porcine gelatin. Values are expressed as mean (standard deviation). For the evaluation of data normality, the Kolmogorov–Smirnov test was used. Plasma concentrations of IL-6, TNF-α, and leptin showed a parametric distribution; therefore, the ANOVA test with Tukey’s post-test was used to determine the significant differences between the evaluated groups. Values of *p* ≤ 0.05 (*** *p* < 0.0001) were considered statistically significant.

Plasma concentrations of IL-6 (Figure 2A) showed a significant reduction (*p* < 0.05) only in the group treated with EPG (test treatment 2), compared to the other evaluated groups. Compared to untreated, standard diet-treated, and CE-treated obesity groups, the mean reduction promoted by EPG treatment was equivalent to 63%.

Concerning plasma concentrations of TNF-α (Figure 2B), it was found that, although no statistically significant difference was observed between the evaluated groups (*p* ≥ 0.05), the animals that received EPG had a lower mean compared to the other groups (Figure 2B).

Regarding leptin (Figure 2C), the group treated with EPG, as observed for IL-6, was the only one that showed a markedly significant decrease in plasma concentrations (*p* ≤ 0.05) compared to the other three groups evaluated, with this average reduction equivalent to 67%.

#### 2.3.3. Weight of Visceral Adipose Tissue

**Figure 3:**
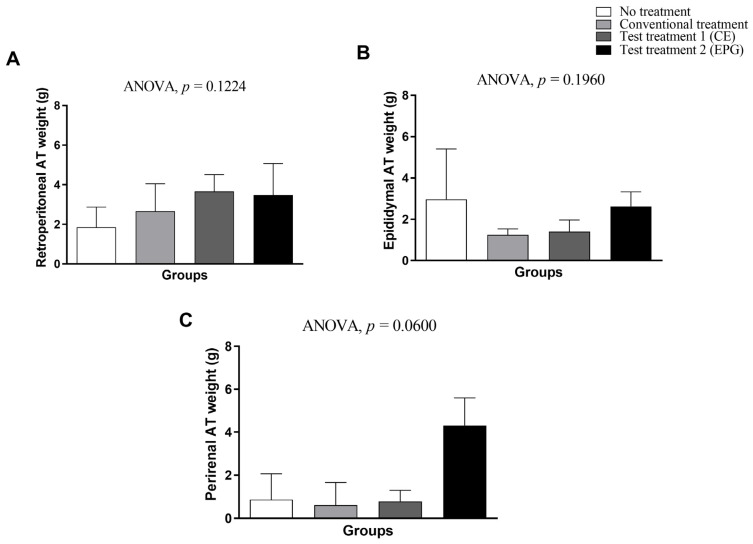
Weight of different types of visceral adipose tissue of adult male Wistar rats (31 weeks), with inflammation induced by HGLI diet, submitted to different treatments and evaluated after ten days of the experiment (11th day). (**A**) Retroperitoneal adipose tissue. (**B**) Epididymal adipose tissue. (**C**) Perirenal adipose tissue. No treatment: HGLI diet + 1 mL of water by gavage; conventional treatment: nutritionally adequate diet (Labina^®^ feed) + 1 mL of water per gavage; test treatment 1: HGLI diet + 1 mL of CE at a concentration of 12.5 mg/kg by gavage; test treatment 2: HGLI diet + 1 mL of EPG at a concentration of 50 mg/kg by gavage; HGLI diet: mixture composed of Labina^®^, condensed milk and sugar (1:1:0.21 *w*/*w*/*w*); HGLI: high glycemic index and high glycemic load diet; CE: crude extract rich in carotenoids from Cantaloupe melons; EPG: crude extract rich in carotenoids from Cantaloupe melons nanoencapsulated in porcine gelatin. Values are expressed as mean (standard deviation). The weights of the retroperitoneal, epididymal, and perirenal adipose tissues showed a parametric distribution, and therefore, the ANOVA test with Tukey’s post-test was used to determine the significant differences. Values of *p* ≤ 0.05 were considered statistically significant.

Regarding the weight of adipose tissues, it is possible to observe that both CE and EPG, respectively, did not affect the total weight of visceral adipose tissues evaluated (*p* > 0.05) (Figure 3).

#### 2.3.4. Histopathological Analysis of Visceral Adipocytes

The histopathological analysis of the adipose tissues of the animals from the four evaluated groups, in general, allowed the observation of the presence of white adipose tissue (WAT), characterized by the presence of unilocular adipocytes.

**Figure 4:**
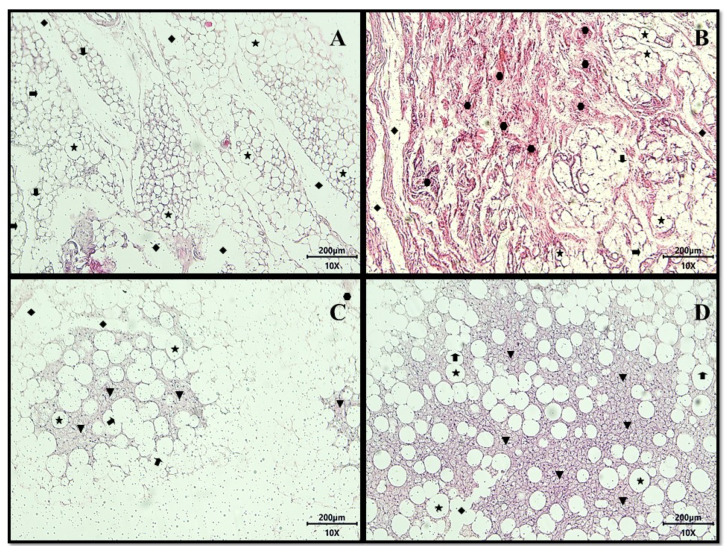
Representative images of histological sections of adipose tissue from adult male Wistar rats (31 weeks), with inflammation induced by the HGLI diet, submitted to different treatments and evaluated after ten days of the experiment (11th day), stained with hematoxylin-eosin (HE) belonging to the following: (**A**) Untreated group. (**B**) Conventional treatment. (**C**) Test treatment 1. (**D**) Test treatment 2. Total magnification 100× (Objective Lens 10×), scale bar: 200 μm. Panoramic evidence of the presence of white adipose tissue consisting of numerous unilocular adipocytes (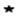) primarily intact, showing focal areas of membrane destruction (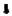), extensive regions of lipolysis channels (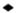), vast areas of fibrosis consisting of dense non-patterned connective tissue in areas of adipocyte destruction (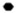), and the presence of the focal regions of multilocular adipocytes (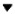). No treatment: HGLI diet + 1 mL of water by gavage; conventional treatment: nutritionally adequate diet (Labina^®^ feed) + 1 mL of water per gavage; test treatment 1: HGLI diet + 1 mL of CE at a concentration of 12.5 mg/kg by gavage; test treatment 2: HGLI diet + 1 mL of EPG at a concentration of 50 mg/kg by gavage; HGLI diet: mixture composed of Labina^®^, condensed milk and sugar (1:1:0.21 *w*/*w*/*w*); HGLI: high glycemic index and high glycemic load diet; CE: crude extract rich in carotenoids from Cantaloupe melons; EPG: crude extract rich in carotenoids from Cantaloupe melons nanoencapsulated in porcine gelatin.

In the slide representing the adipose tissue of the animals in the untreated group, unilocular adipocytes were noted, most with intact structure, but some with focal areas of membrane destruction, lipolysis channels, and focal regions of multilocular adipocytes (Figure 4A).

Concerning the group treated with the nutritionally adequate diet (conventional treatment), the presence of extensive areas of fibrosis formed by dense non-modeled connective tissue (collagen or cell-matrix fibers) observed in regions that had previously shown the destruction of adipocytes indicated an area of improvement in the inflammatory process (Figure 4B).

The animals that received CE treatment (test treatment 1; 12.5 mg/kg of weight) presented, in addition to the previously mentioned characteristics, focal areas of multilocular adipocytes and the presence of regions of fibrosis formed by non-modeled dense connective tissue (Figure 4C).

The group that received the HGLI diet associated with the EPG (test treatment 2; 50 mg/kg of weight) present, in addition to the previously mentioned characteristics, a greater number of focal areas of adipocytes multilocular (Figure 4D).

#### 2.3.5. Measurement of Cytokines (TNF-α and IL-6) in Visceral Adipose Tissue

**Figure 5:**
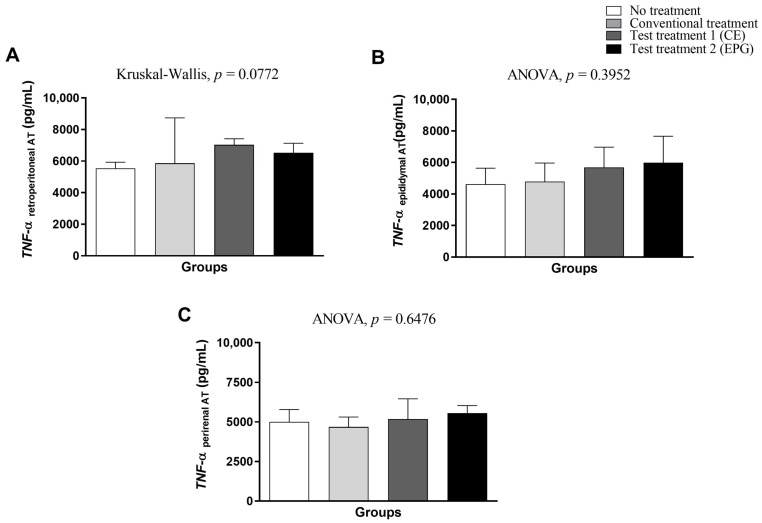
TNF-α concentration in visceral adipose tissue of adult male Wistar rats (31 weeks) with inflammation induced by HGLI diet, submitted to different treatments and evaluated after ten days of the experiment (11th day). (**A**) Retroperitoneal adipose tissue. (**B**) Epididymal adipose tissue. (**C**) Perirenal adipose tissue. No treatment: HGLI diet + 1 mL of water by gavage; conventional treatment: nutritionally adequate diet (Labina^®^ feed) + 1 mL of water per gavage; test treatment 1: HGLI diet + 1 mL of CE at a concentration of 12.5 mg/kg by gavage; test treatment 2: HGLI diet + 1 mL of EPG at a concentration of 50 mg/kg by gavage; HGLI diet: mixture composed of Labina^®^, condensed milk and sugar (1:1:0.21 *w*/*w*/*w*); HGLI: high glycemic index and high glycemic load diet; CE: crude extract rich in carotenoids from Cantaloupe melons; EPG: crude extract rich in carotenoids from Cantaloupe melons nanoencapsulated in porcine gelatin. Values are expressed as mean (standard deviation). The TNF-α concentrations in the epididymal and perirenal adipose tissues showed a parametric distribution; therefore, the ANOVA test with Tukey’s post-test was used to determine the significant differences. The TNF-α concentration of the retroperitoneal adipose tissue showed a non-parametric distribution; therefore, the Kruskal–Wallis test with Dunn’s post-test was used to verify differences between the assessed groups. Values of *p* ≤ 0.05 were considered statistically significant.

Regarding the measurement of TNF-α in visceral adipose tissue (retroperitoneal, epididymal, and perirenal), it is possible to see that test treatments 1 and 2 were not sufficient to provide a statistically significant effect in the reduction in this cytokine than the control groups without treatment and conventional treatment (*p* > 0.05) (Figure 5).

**Figure 6:**
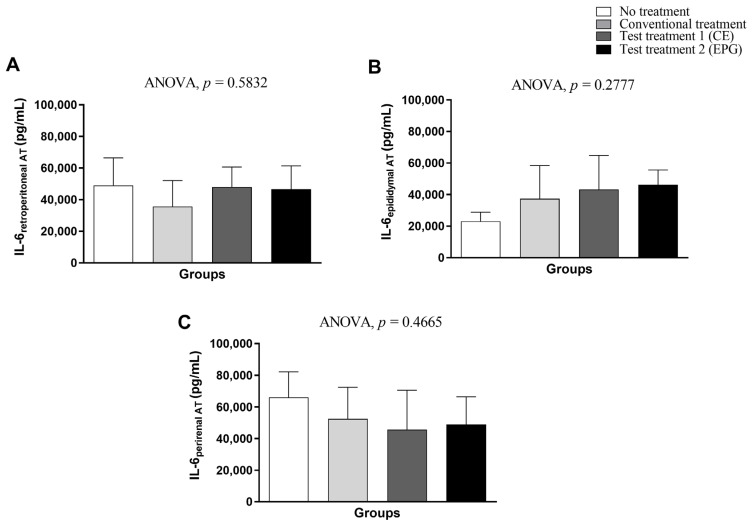
IL-6 concentration in visceral adipose tissue of adult male Wistar rats (31 weeks) with inflammation induced by HGLI diet, submitted to different treatments and evaluated after ten days of the experiment (11th day). (**A**) Retroperitoneal adipose tissue. (**B**) Epididymal adipose tissue. (**C**) Perirenal adipose tissue. No treatment: HGLI diet + 1 mL of water by gavage; conventional treatment: nutritionally adequate diet (Labina^®^ feed) + 1 mL of water per gavage; test treatment 1: HGLI diet + 1 mL of CE at a concentration of 12.5 mg/kg by gavage; test treatment 2: HGLI diet + 1 mL of EPG at a concentration of 50 mg/kg by gavage; HGLI diet: mixture composed of Labina^®^, condensed milk and sugar (1:1:0.21 *w*/*w*/*w*); HGLI: high glycemic index and high glycemic load diet; CE: crude extract rich in carotenoids from Cantaloupe melons; EPG: crude extract rich in carotenoids from Cantaloupe melons nanoencapsulated in porcine gelatin. Values are expressed as mean (standard deviation). The IL-6 concentrations in adipose tissues showed a parametric distribution, and therefore, the ANOVA test with Tukey’s post-test was used to determine the significant differences between the evaluated groups. Values of *p* ≤ 0.05 were considered statistically significant.

Regarding the IL-6 concentration in visceral adipose tissue (retroperitoneal, epididymal, and perirenal), it is also possible to see that both treatments with CE and EPG were not sufficient to provide a statistically significant effect in the reduction in cytokines compared to the control groups without treatment and conventional treatment (*p* > 0.05) (Figure 6).

#### 2.3.6. Determination of Thiobarbituric Acid Reactive Substances (TBARS) in Plasma and Visceral Adipose Tissue

**Figure 7:**
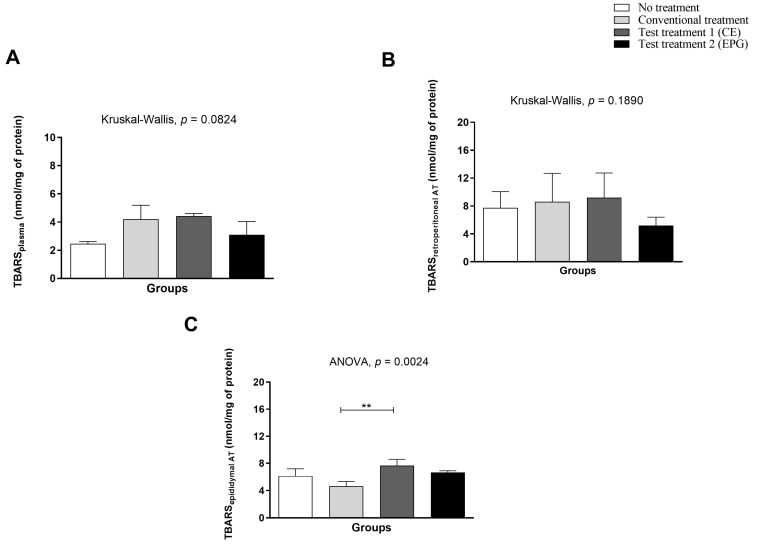
Malondialdehyde in plasma and visceral adipose tissue of adult male Wistar rats (31 weeks) with inflammation induced by HGLI diet, submitted to different treatments and evaluated after ten days of the experiment (11th day). (**A**) Plasma. (**B**) Retroperitoneal adipose tissue. (**C**) Epididymal adipose tissue. No treatment: HGLI diet + 1 mL of water by gavage; conventional treatment: nutritionally adequate diet (Labina^®^ feed) + 1 mL of water per gavage; test treatment 1: HGLI diet + 1 mL of CE at a concentration of 12.5 mg/kg by gavage; test treatment 2: HGLI diet + 1 mL of EPG at a concentration of 50 mg/kg by gavage; HGLI diet: mixture composed of Labina^®^, condensed milk and sugar (1:1:0.21 *w*/*w*/*w*); HGLI: high glycemic index and high glycemic load diet; TBARS: thiobarbituric acid reactive substances; CE: crude extract rich in carotenoids from Cantaloupe melons; EPG: crude extract rich in carotenoids from Cantaloupe melons nanoencapsulated in porcine gelatin. Values are expressed as mean (standard deviation). Plasma and retroperitoneal adipose tissue concentrations showed non-parametric distribution, so the Kruskal–Wallis test with Dunn’s post-test was used to verify differences between the evaluated groups. The concentration in the epididymal adipose tissue showed a parametric distribution; therefore, the ANOVA test with Tukey’s post-test was used to determine the significant differences between the evaluated groups. Values of *p* ≤ 0.05 (** *p* = 0.01) were considered statistically significant.

Regarding the quantification of malondialdehyde and thiobarbituric acid reactive substances (TBARS), it is possible to note that in plasma and retroperitoneal adipose tissue, there was no statistically significant difference between the evaluated groups (*p* > 0.05). In the epididymal adipose tissue, it was possible to observe a significant difference between the conventional treatment and the group of animals treated with CE, the latter being the one with the highest concentration of TBARS (nmol/mg of protein) than the other groups (Figure 7).

#### 2.3.7. Determination of Sulfhydryls in Plasma and Visceral Adipose Tissue

**Figure 8:**
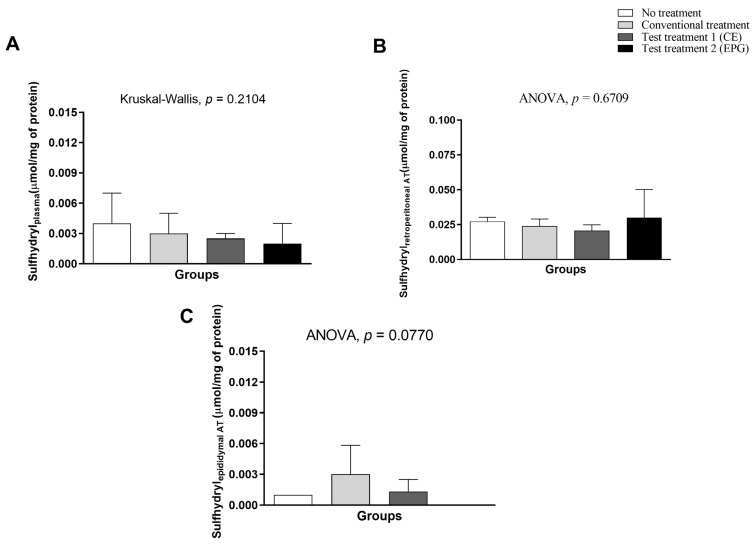
Sulfhydryls in plasma and visceral adipose tissue of adult male Wistar rats (31 weeks) with inflammation induced by HGLI diet, submitted to different treatments and evaluated after ten days of the experiment (11th day). (**A**) Plasma. (**B**) Retroperitoneal adipose tissue. (**C**) Epididymal adipose tissue. No treatment: HGLI diet + 1 mL of water by gavage; conventional treatment: nutritionally adequate diet (Labina^®^ feed) + 1 mL of water per gavage; test treatment 1: HGLI diet + 1 mL of CE at a concentration of 12.5 mg/kg by gavage; test treatment 2: HGLI diet + 1 mL of EPG at a concentration of 50 mg/kg by gavage; HGLI diet: mixture composed of Labina^®^, condensed milk and sugar (1:1:0.21 *w*/*w*/*w*); HGLI: high glycemic index and high glycemic load diet; CE: crude extract rich in carotenoids from Cantaloupe melons; EPG: crude extract rich in carotenoids from Cantaloupe melons nanoencapsulated in porcine gelatin. Values are expressed as mean (standard deviation). Plasma concentrations showed non-parametric distribution, so the Kruskal–Wallis test with Dunn’s post-test was used to verify the difference between the evaluated groups. The concentration in adipose tissues showed a parametric distribution; therefore, the ANOVA test with Tukey’s post-test was used to determine the significant differences between the evaluated groups. Values of *p* ≤ 0.05 were considered statistically significant.

Concerning sulfhydryls, it is possible that both in the plasma and in the retroperitoneal and epididymal adipose tissues, there was no statistically significant difference between the evaluated groups (*p* > 0.005) (Figure 8).

#### 2.3.8. Determination of the Enzymatic Activity of Superoxide Dismutase (SOD) in Plasma

**Figure 9:**
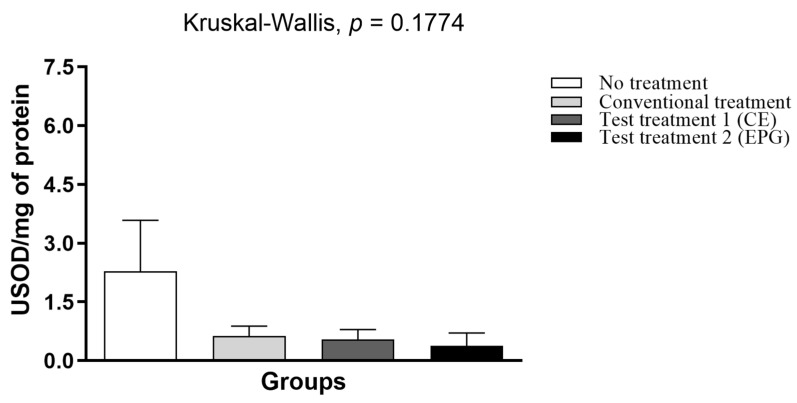
Superoxide dismutase (SOD) activity in the plasma of adult male Wistar rats (31 weeks) with inflammation induced by the HGLI diet, submitted to different treatments and evaluated after ten days of the experiment (11th day). No treatment: HGLI diet + 1 mL of water by gavage; conventional treatment: nutritionally adequate diet (Labina^®^ feed) + 1 mL of water per gavage; test treatment 1: HGLI diet + 1 mL of CE at a concentration of 12.5 mg/kg by gavage; test treatment 2: HGLI diet + 1 mL of EPG at a concentration of 50 mg/kg by gavage; HGLI diet: mixture composed of Labina^®^, condensed milk and sugar (1:1:0.21 *w*/*w*/*w*); HGLI: high glycemic index and high glycemic load diet; CE: crude extract rich in carotenoids from Cantaloupe melons; EPG: crude extract rich in carotenoids from Cantaloupe melons nanoencapsulated in porcine gelatin. Values are expressed as mean (standard deviation). Plasma concentrations showed non-parametric distribution, so the Kruskal–Wallis test with Dunn’s post-test was used to verify the difference between the evaluated groups. Values of *p* ≤ 0.05 were considered statistically significant.

Based on the results obtained (Figure 9), it is possible to observe that there was no statistically significant difference in SOD (*p* > 0.05). However, except for the untreated group, all others had reduced enzymatic activity in plasma.

## 3. Discussion

Previous in vivo and in vitro studies conducted by Medeiros et al. [16] and Oliveira et al. [15] pointed out, respectively, the EPG’s safety and lack of toxicity and the increase in the antioxidant potential. Furthermore, Gomes [17] found the ability of EPG to increase hepatic retinol reserves in an animal model with chronic inflammation without promoting hepatic alterations, suggesting that there may have been an increase in the absorption of carotenoids in the intestines of animals. This situation probably occurred due to the increased solubility and stability of carotenoids, demonstrated in previous studies [14,15], which can positively affect the provitamin A activity promoted by α and β-carotene in EPG.

Thus, continuing the investigations to evaluate the potential of the nanoformulation (EPG) in the present study, the effect of the nanoencapsulation of the extract rich in carotenoids from Cantaloupe melons (*Cucumis melo* L. var cantalupensis) was evaluated in a preclinical model of chronic systemic inflammation and excess adiposity induced by the HGLI diet.

Because of the data exposed, it is essential to emphasize that, based on previous studies, a ratio of 1:80 *w*/*w* (CE: encapsulating agent) was used to produce EPG. Therefore, the group treated with EPG (50 mg/kg) received an amount of CE 20 times lower (0.62 mg/kg) than the group treated with non-encapsulated CE (12.50 mg/kg), reinforcing the justification for potentiating the bioactive properties of CE after nanoencapsulation.

In this study, the EPG appeared as spherical particles with a smooth surface, without pores, and with a physical size of less than 100 nm. In addition, the laser diffraction technique confirmed that homogeneous nanoparticles were formed. In this perspective, comparing the results obtained in this study with published studies [14,15,17], it was possible to infer that the present particle presented similar physicochemical characteristics, suggesting that, in the present study, there was the encapsulation of the crude extract of carotenoids extracted from Cantaloupe melons (EPG). In addition, the previously described characteristics were maintained, which directly influence the EPG functionalities, providing an indication of standardization and good reproducibility in obtaining a new batch of EPG and, therefore, enabling new applications of this material, such as, for example, in the present preclinical study in an animal model.

Oliveira et al. [15] observed that the nanoformulation without carotenoids did not exhibit antioxidant activity. However, an increase in antioxidant activity was observed evaluating CE after its extraction from the interior of EPG (after encapsulation). Thus, it was proved that the encapsulating agents were not responsible for the antioxidant activity but the nanoencapsulation of the CE with these agents. Thus, the authors observed that the CE was well protected, which made it difficult to interact with free radicals present in the reaction medium, requiring the extraction of CE from the interior of the particles to promote antioxidant activity. Therefore, the nanoformulation without CE was not evaluated in the present study.

In the preclinical study, although there was no significant difference (*p* > 0.05), the group treated with EPG showed negative dietary intake and caloric efficiency variations. Furthermore, the group that received the EPG was the only one in which only weight loss was observed in all animals during the treatment period. On the other hand, the animals in the untreated group showed the greatest gains for the parameters already mentioned. Considering the variation in body weight was also negative for the group treated with EPG (Table 2), this weight loss may be related to reduced food intake. Because of that the EPG had, in its composition, β-carotene in the majority [14], it is suggested that this carotenoid may be directly influencing these presented results since the literature shows that the supplementation of these bioactive compounds acts on the dietary intake reduction and consequently in the body weight reduction [18].

Furthermore, in the present study, it was observed that only the EPG could reduce plasma leptin concentrations close to those observed in eutrophic animals. These data indicated that the EPG would promote an improvement in the uptake of leptin, which the direct negative regulation of leptin gene expression in adipose tissue can explain. With that, there was the re-establishment of satiety control functions and, consequently, the decrease in body weight and the modulation of adipose tissue [17,19,20].

In situations of obesity, “leptin resistance” occurs, in which the circulating concentrations of this hormone increase. However, because it cannot act in the cells, there is a loss of ability to perform in the control of satiety. In the literature, studies describe that supplementation with carotenoids improves the inflammatory response and reduces hyperleptinemia. In addition, it promotes satiety, leading to a smaller variation in weight gain [19,20], corroborating the results obtained in this study.

Additionally, in obesity, body fat deposits are increased, with a consequent increase in the expression and secretion of adipokines proportionally to the greater volume of adipose cells. The fat deposits, namely, visceral, abdominal subcutaneous, femoral gluteal subcutaneous, and intramuscular adipose tissue, have differentiated metabolic and endocrine levels and may therefore be interfering specifically in the processes inherent to body adiposity in obesity. In addition, in excess weight, there is hypertrophy of the adipose tissue, causing an increase in the size of unilocular adipocytes. Hence, cytokine secretion, immune cell infiltration, systemic inflammation, and fibrosis are altered. However, when subjected to certain stimuli, there is a change in the adipogenesis process, which is driven by transcription factors such as PPARγ responsible for the thermogenic brown or beige phenotype, resulting in the appearance of beige adipocytes [21].

In this perspective, and based on the literature, the findings observed in the present study through histological analyses may be related to the remodeling of the adipose tissue since it was possible to observe an increase in focal areas of multilocular adipocytes in the animals in the group treated with EPG. On the other hand, a reduction in white adipocytes has been directly associated with reductions in serum IL-6 and leptin [22]. From this angle, the EPG may have presented its anti-inflammatory action related to the modulation of adipose tissue, acting on adipocyte differentiation, which could be related to adipogenesis and/or differentiation processes.

Amengual et al. [23] evaluated the effect of β-carotene supplementation, but in a more extended period and higher concentration (150 mg/kg of diet for 14 weeks), and observed that, although it did not significantly affect the final body weight of the animals, β-carotene acted by reducing body adiposity (28%), plasma leptin, PPARγ expression (resulting in a decrease in adipogenesis), and the number of unilocular adipocytes.

In this study, considering the data obtained, it is believed that one of the forms of action of the nanoformulation on weight, leptin concentration, and adipose tissue remodeling would be sustained by the provitamin A activity of the carotenoids present in EPG, suggested according to the findings from the study by Gomes et al. [17]. Furthermore, from this perspective, studies show a direct correlation between the excess adiposity observed in obesity and the reduction in plasma concentrations of vitamin A and carotenoids with provitamin A action, reducing bioavailability and activity on oxidative stress and inflammation [6]. Possibly, nanoencapsulation promoted greater protection of carotenoids against the action of enzymes and acid pH in the stomach, facilitating delivery to the target site (intestine) and conversion of carotenes (α and β-carotene) to retinol in the liver.

It is also known that treatment with EPG could be causing a significant reduction in M1-type macrophages, responsible for the secretion of pro-inflammatory cytokines (TNF-α and IL-6) and reactive oxygen species. On the other hand, promoting an increase in M2-type macrophages, responsible for the secretion of anti-inflammatory cytokines (IL-10, IL-4, and TGF-β) and blocking the action of M1 macrophages, improving inflammation [24]. Thus, it would reduce the expression of genes related to adipogenesis, such as PPARγ and transcription factor sterol regulatory element binding protein-1c (SREBP-1c), and increase the expression of uncoupling proteins 1 and 2 (UCP1 and UCP2, respectively), responsible for the beige of white adipose tissue by promoting thermogenesis [25,26,27,28].

From this perspective, treatments with CE and EPG could be acting on monocytes, as highlighted by Lee, Yoo, and Lee [24], who observed that mice that received treatment with melon (*Cucumis melo* L.—in this study, mice were administered an extract of *Cucumis* (20–40 mg/kg) orally twice a week) showed a significant reduction in classic “pro-inflammatory” monocytes. These are responsible for the secretion of inflammatory cytokines such as TNF-α and IL-1β and their decrease. On the other hand, they are responsible for the increase in IL-10. Thus, this study pointed out that *Cucumis melo* can act both on plasma monocytes with a systemic effect and locally on macrophages in adipose tissue.

Additionally, the provitamin A action is directly related to inhibiting adipogenesis, suppressing the gene expression of PPARα, PPARγ, and RXR, modulating the adipose tissue by inducing WAT darkening, promoting the appearance of multilocular and thermogenic adipocytes, controlling adiposity and inflammation in adipocytes [25]. In addition, the provitamin A action is also associated with the NF-κB reduction, the infiltration of macrophages in the adipocytes, and reduced excess adipose tissue in the animals [18,29]. However, in the present study, it is still impossible to state what caused this change in the adipose tissue in the animals treated with EPG.

Kim et al. [30] evaluated the effect of carotenoids obtained from the extraction of red paprika (*Capsicum annuum* L.; a high-fat diet containing 10% (*w*/*w*) powdered red paprika) and commercial capsanthin (a high-fat diet containing 0.025% (*w*/*w*) powdered capsanthin) for eight weeks, in the adipose tissue of mice with obesity. They observed that carotenoids reduced adipocyte hypertrophy by acting in the adipogenesis process, improving plasma concentrations of leptin. Although it is impossible to compare the concentrations administered to the animals with the present study, it is possible to observe that the duration time was longer than that of CE and EPG administration.

It is known that higher plasma concentrations of leptin, IL-6, and TNF-α are directly associated with the expansion of adipose tissue and lipolysis [3]. Therefore, although it is not clear how, in this study, a lower plasma concentration of these inflammatory cytokines related to EPG treatment was observed, another possibility of explaining the observed systemic anti-inflammatory effect would be through the antioxidant activity of carotenoids. This possibility is supported by Oliveira et al. [15], which found that nanoencapsulation promoted the potentiation of the in vitro antioxidant activity of CE extracted from EPG. The potentiation of the antioxidant action through nanoencapsulation is already well documented in the literature [31].

Studies by the NutriSBioativoS group showed that new chemical interactions emerge (such as hydrogen interactions and hydrophobic and Van der Waals forces) when encapsulating these bioactive compounds, increasing surface area, dispersibility in water, stability, and higher antioxidant potential. This fact was proven in vitro, making it possible to visualize greater stability against different storage conditions (25 °C (dark) and 5 °C (light and dark)) and increased antioxidant activity of the extract of Cantaloupe melon carotenoids after encapsulation in porcine gelatin [15,31].

Based on this reasoning related to the possible ways of action, it is possible to visualize that the treatment with EPG was not enough to act in the local inflammatory process, considering that it did not significantly reduce the cytokines in the visceral adipose tissue. EPG promoted a discreet reduction in oxidative stress in this organ and the plasma. The data suggest that the nanoformulation, containing a 20 times lower dose of CE, may be acting in the partial neutralization of TBARS in the plasma and adipose tissues, reducing the observed oxidative damage.

Still, concerning the antioxidant activity in organs, it was possible to observe that, in the retroperitoneal adipose tissue of the animals that received the test treatment 2 (EPG), there was a slight reduction in oxidative stress, considering that there was a reduction in lipid peroxidation, indicated by a decrease in TBARS and an increase in antioxidant defenses, represented by the rise in sulfhydryls. This fact suggests that oral EPG supplementation, even using a lower CE concentration, reduced this biomarker of oxidative stress and probably also acted to improve oxidative damage, although not significantly.

From this perspective, it was observed that the results obtained from SOD show that the treatment with EPG acted by decreasing the action of this enzyme responsible for the antioxidant defense. According to the literature, this fact can be explained by the considerable presence of carotenoids in the nanoparticle, which, after the encapsulation process, had their action and efficiency enhanced, as documented in previous studies carried out with this same nanoparticle [15]. EPG probably assumed the role of a free radical scavenger, acting in the first step of the detoxification chain of superoxide radicals, producing hydrogen peroxide, which perhaps, due to its high concentration, could be promoting catalase activity and activating the second step of the detoxification chain, resulting in water and oxygen [32,33,34]. With that, the EPG, even containing low CE concentrations, would be improving the detoxification of free radicals, reducing their concentration and possibly acting as a mimetic SOD in the defense action against the damage associated with oxidative stress. However, to confirm this assumption, catalase activity needs to be performed, but it was not possible, which is a limitation of this study.

In this context, one of the possible pathways involved in this antioxidant action by the action of carotenoids would be the activation of NF-κB (nuclear factor Kappa B (P65/P50)) in adipocytes and the reduction in macrophage infiltration in adipose tissue [18]. In a state of obesity, as in the experimental model used in this study, there is a greater expression of inflammatory markers, which activate the IKK complex (IkB kinase (IKKβ and IKKα)). Thus, this initiates the phosphorylation-dependent degradation of IκBα, resulting in the translocation of NF-κB to the nucleus and the upregulation necessary for increased synthesis of TNF-α and IL-6 [35]. Additionally, data related to plasma cytokines confirm these increases in TNF-α and IL-6 in animals with obesity and without treatment.

However, in the present study, there was no significant difference in the concentration of these cytokines (TNF-α and IL-6) in visceral adipose tissue. Because of the data obtained in this study, as future perspectives, other analyses related to the expression of genes associated with the activation of NF-Κb or the IKK complex and also of IL-6, leptin, and TNF-α can be performed, in addition to analyzing other inflammatory cytokines, such as IL1-β and IL-8. Furthermore, those related to the suppression of gene expression of PPARα, PPARγ, and RXR, and also analyses in adipocyte cell culture, may explain the molecular mechanisms that are impacting the remodeling of adipose tissue and, consequently, that are involved in the systemic reduction in these cytokines, among others, and can be used to clarify the related mechanisms.

One of the limitations of the literature lies in the low number of studies that evaluate the effect of encapsulated carotenoids in preclinical or clinical studies. The present study showed that nanoencapsulation of carotenoids is a promising tool to protect and enhance the action of these pigments. Mainly, it provided a direction for new perspectives of studies using the EPG, considering that it proved to be a possibility of use as a bioactive potential in treating diseases related to excess adipose tissue and inflammation.

## 4. Materials and Methods

### 4.1. Materials

The melons used in this study were of the commercial type of Cantaloupe (*Cucumis melo* L.) acquired in local commerce in the city of Natal (RN) and registered in the National System of Genetic Heritage Management and Associated Traditional Knowledge (SISGEN) under the number A5A85DF. The materials used in the nanoencapsulation step were Lizza^®^ brand soybean oil (also purchased locally), porcine gelatin (type A), and Tween 20 surfactant, obtained from Sigma-Aldrich. The β-carotene standard used in Ultra Performance Liquid Chromatography (UPLC (Shimadzu, Kyoto, Japan) to characterize the extract was obtained from Sigma^®^.

### 4.2. Obtaining, Characterization, and Nanoencapsulation of the Crude Extract Rich in Carotenoids from Cantaloupe Melons

The procedures for obtaining, characterization (UV-Vis absorption spectrophotometry and Ultra Performance Liquid Chromatography), and nanoencapsulation of the extract rich in carotenoids from Cantaloupe melons with subsequent drying by lyophilization were carried out according to Medeiros et al. [14]. The materials obtained were named crude extract rich in carotenoids from Cantaloupe melons (CE) and crude extract rich in carotenoids from Cantaloupe melons nanoencapsulated in porcine gelatin (EPG) [14].

To obtain CE, Cantaloupe melon pulp was dried (55 °C/24 h) to obtain pulp flour, which was subjected to maceration using 95% *v*/*v* ethanol (1:4 *w*/*v*) until color loss. The ethanolic extract obtained was partitioned using hexane PA (1:1 *v*/*v*) and 10% sodium chloride (NaCl) solution (1:10 *v*/*v*) until the hexane phase became colorless. The solvent was eliminated with the aid of rotavapor (Buchi—R-215) under low pressure at a temperature of 27 °C, followed by lyophilization (−57 °C/43 mmHg) [14].

For the synthesis of EPG, the technique of O/W emulsification was used, followed by dispersion in an aqueous phase containing an encapsulating agent (porcine gelatin), with subsequent drying by lyophilization [14]. Thus, two aqueous phases were previously prepared: FA 1 (90 mL), containing 1.5% Tween 20 (*w*/*v*), which was solubilized in distilled water, and FA 2 (100 mL), including 4% porcine gelatin (*w*/*v*) and 1.5% Tween 20 (*w*/*v*), both solubilized in distilled water. The oil phase was prepared using 0.5% CE (*w*/*v*), which was solubilized in soybean oil (10 mL). Both the first emulsion (FA1 + oil phase) and the dispersion (emulsion 1 + aqueous phase 2) were obtained using an ultra-disperser (Ultra-Turrax, IKA^®^T18 basic (IKA® Werke Gmbh & Co. KG, Staufen, Germany)) at 17,000 RPM for 10 min. Subsequently, lyophilization was performed (LioTop L101) at a temperature of −57 °C and a pressure of 43 mmHg.

#### 4.2.1. Characterization of the Nanoparticles (EPG)

The nanoparticles were characterized by Scanning Electron Microscopy (SEM), laser diffraction, and Fourier Transform Infrared Spectrophotometry (FTIR) methods, according to established protocols [14,16].

The morphology was investigated using the SEM-FEG ZEISS (AURIGA). For this, the nanoparticles were suspended in acetone, being later dripped onto silicon plates fixed with carbon tape in the stub. Micrographs were obtained in high vacuum, 2–3 kV tension, at different magnifications, without gold plating, according to Medeiros et al. [14].

To evaluate the diameter of the particles, first, the nanoparticles were deagglomerated using formaldehyde, as proposed by Medeiros et al. [14]. Subsequently, they were dispersed in water, inserted into a glass cuvette, and read at 10 runs/min on the NanoBrookZetaPlusZeta Potential Analyzer (Brookhaven Instruments, Holtsville, NY, USA), Brookhaven-Instruments software—ZetaPALS ParticleSizing Software version number 7.10. It should be noted that the experiment was performed in triplicate.

Chemical interactions between EPG constituent materials were evaluated using FTIR. CE, porcine gelatin, Tween 20, and EPG were homogenized separately with potassium bromide (KBr), macerated, and pressed to form tablets. They were evaluated (SHIMADZU IR tracer-100, (Shimadzu, Kyoto, Japan)) in the range of 400–4000 cm^−1^, with a scan of 32 and a resolution of 4 cm^−1^ [14].

#### 4.2.2. Determination of Incorporation Efficiency (IE) (%)

The incorporation efficiency (IE) was determined according to Medeiros et al. [14]. For this, 150 mg of EPG were dispersed in hexane (1 mL), sonicated (Ultra cleaner 1650 Unique^®^) for 3 min, and centrifuged (Micro Centrifuge 6000 RPM HT^®^) at 947.52× *g* for 20 min) to promote the separation of carotenoids in the supernatant. The procedure was repeated in triplicate until color exhaustion. The absorbance of the supernatant was measured in a spectrophotometer (Bel photonics 1105, 450 nm) to determine the concentration of carotenoids present in EPG, using the equation of the line previously obtained, using hexane as solvent.

Finally, the equation proposed by Hu et al. [36] (Equation (1)) was used to determine the content of crude extract-rich carotenoids encapsulated inside particles.

IE (%) = (carotenoids in particles/total carotenoid extract used) × 100(1)

### 4.3. In Vivo Study

The in vivo investigation was carried out at the Potiguar University Animal Facility (UnP) following the ARRIVE guidelines (https://arriveguidelines.org/, accessed on 26 July 2018) and approved by the Ethics in Animal Use Committee (CEUA-UnP) under the protocol of approval No. 019/2017.

Wistar rats (350–450 g), adults of 21 weeks of age with chronic systemic inflammation induced by a high glycemic index and high glycemic load diet (HGLI diet) from UnP, were used. Inflammation caused by the HGLI diet was studied by Luz et al. [37], who evaluated the inflammatory effect of this diet in Wistar rats and observed that, when consumed for 17 weeks, it led to adipocyte hypertrophy, greater fat deposition in the liver and pancreas, greater gene expression and plasma concentration of TNF-α, alteration, and inflammation in the intestinal epithelium compared to animals that consumed a nutritionally adequate diet. The animals were randomly distributed in individual cages under standard light (12 h light/12 h dark), temperature (23–25 °C), water, and food ad libitum.

#### 4.3.1. Diets and Experimental Groups

The experimental diets used in the in vivo experiment were the standard diet (Labina^®^), a commercially available nutritionally adequate diet (Paulínia, São Paulo, Brazil), and the HGLI diet [37], as an inducer of chronic systemic inflammation, containing condensed milk, sugar, and Labina^®^ diet as ingredients. For every 100 g, 45.2 g of previously ground Labina^®^ feed was supplemented with 9.6 g of refined sugar and 45.2 mL of condensed milk. After being homogenized, the mixture was baked at 180 °C for approximately 40 min.

Initially, the animals were randomly allocated to individual cages and underwent five days of adaptation to establish a pattern of food consumption. Subsequently, the animals in the four groups received the diets and respective treatments for 11 days.

The conventional treatment group received a nutritionally adequate diet (Labina^®^ chow) and water by gavage (1 mL/day) (n = 5). The other groups were named test treatment 1 (HGLI diet and CE) (n = 5) and test treatment 2 (HGLI diet and EPG) (n = 5), being administered, by gavage, 12.5 mg/kg and 50 mg/kg (1 mL/day), respectively, in addition to an untreated group, which received only the HGLI diet.

The doses were established in vitro [16] based on previous evaluation of the amount necessary to inhibit by 50% (IC50) the activity of ABTS radicals (2,2′-azino-bis (3-ethylbenzothiazoline) 6-sulfonic acid) and DPPH (2,2-diphenyl-1-picrylhydrazyl). Therefore, 50 mg/kg of EPG showed the same radical inhibition effect observed for 12.5 mg/kg CE. EPG contains a smaller amount of CE (0.62 mg of CE in 50 mg of EPG), 20 times less than that administered to the group that received test treatment 1 (12.5 mg/kg). In addition, EPG concentration administered in Wistar rats was also based on the study by Pinzón-García et al. [38]. It is important to emphasize that previous in vivo and in vitro studies conducted by Medeiros et al. [16] and Oliveira et al. [15] pointed out, respectively, the EPG’s safety and lack of toxicity and the increase in the antioxidant potential.

#### 4.3.2. Food Consumption, Caloric Intake, Caloric Efficiency, and Body Weight

During the experiment (11 days), the animals received previously weighed portions of their respective diets. For this calculation, the initial consumption (day 1) was considered, after adaptation and establishment of the individual consumption pattern, and after the final consumption (day 11), thus obtaining the delta (Δ) of dietary consumption (Equation (2)) or, more precisely, the variation in dietary intake. Residual spillage in cages [39] was not considered. More specifically, the values were calculated for each animal, obtaining each evaluated group’s mean (SD).

ΔDietary intake = final weight of consumed diet − initial weight of consumed diet(2)

Concerning weight, the animals were weighed individually on the same days as the assessment of food consumption, at the beginning (day 1) and the end (day 11), obtaining the mean and standard deviation (SD) per group. Thus, the delta (Δ) of weight (Equation (3)) or, more precisely, the variation in body weight was obtained. In addition, the average weight loss and gain (g) based on the number of animals from the same group that lost or gained weight was also evaluated.

ΔBody weight = final body weight − initial weight(3)

The caloric efficiency was obtained below (Equations (4) and (5)), with 4.184 equivalent to the caloric conversion in kilojoules (KJ).

Caloric intake (KJ) = Final dietary intake (Kcal) × 4.184(4)

Caloric efficiency = Caloric intake (KJ) ÷ ∆ body weight(5)

Weight gain and loss (g) were obtained based on Equation (6). This calculation was performed for each rat, determining the mean (SD) for each group evaluated.

Weight loss/gain (g) = final weight − initial weight(6)

#### 4.3.3. Plasma Concentrations of IL-6, TNF-α, and Leptin in an Experimental Model

On the 11th day of the experiment, the animals in the different groups were fasted (8 to 12 h) and subsequently anesthetized (250 mg of tiletamine hydrochloride and 250 mg of zolazepam hydrochloride) for blood collection by cardiac puncture after longitudinal cut made from the base of the abdomen to the outer part, with the aid of surgical scissors. Trained and licensed veterinarians performed all anesthesia, euthanasia, and blood and organ collection procedures.

Then, the blood was collected in tubes with EDTA and destined for the evaluation of plasmatic TNF-α, IL-6, and leptin, being previously centrifuged (3345 RPM/10 min at 4 °C) to separate the plasma, which was submitted to analysis, using the Rat Leptin ELISA kits (Millipore^®^—EZRL—83K (Thermo Fisher Scientific, Waltham, MA, USA), Quantikine Rat TNF-α Immunoassay, and Quantikine Rat IL-6 Immunoassay (R&D Systems, São Paulo, Brazil), following the manufacturer’s instructions. All analyses were performed by a professional unaware of the type of treatment received by each animal.

As reference standards of normality, the values obtained from healthy, eutrophic (320–380 g) adult Wistar rats acclimatized in the same conditions of this study and fed with a nutritionally adequate diet (Labina^®^ diet) were considered to be IL-6 1.74 (0.11) pg/mL, TNF-α 3.26 (0.62) pg/mL, and Leptin 1.76 (0.21) ng/mL.

#### 4.3.4. Visceral Adipose Tissue Weight

The animals’ visceral adipose tissue (perirenal, retroperitoneal, and epididymal) after euthanasia was also collected after a longitudinal cut made from the base of the abdomen to the outside, with the aid of surgical scissors, exposing the entire abdominal and thoracic cavity of the animals.

The tissues were collected by qualified and trained professionals, guaranteeing the complete extraction of all tissues. After collection, the organs were weighed on an analytical scale (Biopreciosa, FA-2104N, Paraná, Brazil) with a precision of 0.0001 g, thus obtaining the weight of each tissue in grams (g).

#### 4.3.5. Histopathological Analysis of Visceral Adipocytes

After determining the weight, the collected adipose tissues were placed individually in sterile plastic pots with lids containing 4% formaldehyde (*v*/*v*), separated and previously identified.

Subsequently, representative fragments obtained by uniform, systematic, and random sampling of adipose tissues were dehydrated in an increasing alcoholic series (70%, 80%, and 90% *v*/*v*), cleared in xylol, impregnated, and embedded in histological paraffin.

The blocks were sectioned in a microtome (Leica RM2235, Buffalo Grove, IL, USA), obtaining thin paraffin slices, showing the longitudinal and transversal sections of the adipose tissue, with 5 μm of thickness. The cuts were placed on a glass slide, deparaffinized, rehydrated, stained with hematoxylin and eosin, covered with a cover slip, and analyzed in an optical microscope B800 (Optika, Pontenarica, Italy), obtaining three slides with three sections/each.

The images were captured with an Optikam PRO6 digital camera (Optika, Pontenarica, Italy) coupled to the microscope mentioned above, using a 10× magnification. Detailed descriptions from the analyses were tabulated for this purpose. The diagnostic reading of the slides was performed blindly by a pathologist in the Laboratory of Histological Techniques in the Department of Morphology of the Biosciences Center of UFRN.

#### 4.3.6. Cytokine Concentration (TNF-α and IL-6) in Visceral Adipose Tissue

The concentrations of interleukins 6 (IL-6) and Tumor Necrosis Factor-alpha (TNF-α) in visceral adipose tissue (perirenal, retroperitoneal, and epididymal) were measured using the enzyme-linked sandwich immunosorbent assay technique (enzyme-linked immunosorbent assay/ELISA), using protocols from R&D kits (Minneapolis, MN, USA).

The adipose tissue was homogenized in phosphate buffer pH 7.4 in a proportion of 1:5 (*v*/*v*) for TNF-α and 1:10 (*v*/*v*) for IL-6, which were centrifuged at 4285 RPM and 4 °C for 10 min (Novatécnica NT 805). The supernatant (100 μL) was used to measure the markers by reading their absorbance at 450 nm (Mindray MR-96A).

The results were interpolated into a standard curve for each marker, obtaining for IL-6 the curve interval from 125 to 8000 pg/mL with the equation of the straight line equivalent to y = 0.0003x + 0.0691 (R^2^ = 0.9998) and, for TNF-α, the curve interval from 62.5 to 4000 pg/mL, with the equation of the straight line equivalent to y = 0.0003x + 0.0978 (R^2^ = 0.9982).

#### 4.3.7. Quantification of Thiobarbituric Acid Reactive Substances (TBARS) in Visceral Adipose Tissue and Plasma by the Thiobarbituric Acid (TBA) Method

The adipose tissues (retroperitoneal and epididymal) were fragmented, weighed (120–200 mg/organ), and stored on ice. The tissue fragments were diluted in KCl (1.15%) in the proportion 1:20 *w*/*v* (tissue:reagent), homogenized, and the supernatants were separated and prepared according to De Souza et al. (2018) [40]. An aliquot of 150 μL of the supernatant was pipetted (in duplicate) into the 96-well plate, and the absorbances were read at 532 nm [40].

Plasma aliquots (250 μL) were centrifuged (12,060 RPM, 4 °C, 10 min), pipetted into Eppendorf containing 10 μL of BHT (4.5 Mm) and 200 μL of trichloroacetic acid (TCA) (30% *w*/*v*) [40]. They were heated in a water bath (90–95 °C) for 15 min and centrifuged again (12,552 RPM, at room temperature, 2 min), removing 400 μL of the supernatant, placing it in cryovials and adding 400 μL TBA 0.73% (*w*/*v*). A total of 200 μL of the supernatants were pipetted (in duplicate) into the 96-well plate, followed by an absorbance reading at 540 nm [40].

#### 4.3.8. Protein Quantification in Tissue and Plasma Samples

Proteins in the plasma and adipose tissue were quantified using the bicinchoninic acid (BCA) method, according to the manufacturer’s instructions (Thermo Fisher Scientific, Waltham, MA, USA).

#### 4.3.9. Determination of Sulfhydryl in Plasma and Visceral Adipose Tissue

The concentration of sulfhydryl groups was determined using the DTNB molar extinction coefficient, corresponding to 14.1 M^−1^ cm^−1^, with the results expressed in μmol/L: [ABS/0.063] = Normalized by protein concentration [41,42,43].

#### 4.3.10. Determination of the Enzymatic Activity of Superoxide Dismutase (SOD) in Plasma

Briefly, the plasma samples were centrifuged (12,060 RPM, 10 min, 4 °C) and diluted in saline solution at a 1:80 ratio (*v*/*v*), using increasing amounts of the dilute (named 10, 25, and 50): 10 (10 μL of sample + 10 μL of adrenaline (kept at 4 °C) + 980 μL of bicarbonate/sodium carbonate buffer pH 10.25), 25 (25 μL of sample + 10 μL of adrenaline (kept at 4 °C) + 965 μL of bicarbonate buffer/sodium carbonate pH 10.25), and 50 (50 μL of sample + 10 μL of adrenaline (kept at 4 °C) + 940 μL of bicarbonate/sodium carbonate buffer pH 10.2) [44]. To remove possible biases, a control (called baseline) consisted of 10 μL of adrenaline and 990 μL of sodium bicarbonate/sodium carbonate buffer pH 10.2 [44].

The analyses were performed in duplicate, and the absorbance was determined at 480 nm in a maximum time of 10 min (with a reading interval of 1 min). The delta was calculated according to Equation 7, and the result was expressed in SOD/g protein [44].

∆ = decrease in absorbance from time 4 min − peak absorbance from baseline(7)

### 4.4. Statistical Analyses

Simple and random sampling was used for the experimental study’s sample size (n) [45]. An anticipated variation coefficient of 10% was adopted with an error probability of less than 5% and a power of 90%, corresponding to an n of 4.4 animals, 5 animals per study group. Thus, a physiologically significant difference in the evaluated parameters was assumed when the treatment promoted an effect greater or equal to 25%, according to the 3Rs principles (replacement, reduction, and refinement). It should be noted that there were no losses in the experimental study.

Data referring to inflammatory parameters were expressed as mean and standard deviation. Caloric intake, plasma cytokines (IL-6, TNF-α, and leptin), retroperitoneal, epididymal, and perirenal adipose tissue weights, as well as TNF-α in epididymal and perirenal adipose tissue, IL-6 in the three adipose tissues evaluated, the concentration of malondialdehyde in the epididymal adipose tissue, and the concentration of sulfhydryls in the retroperitoneal and epididymal adipose tissues showed a parametric distribution and, therefore, were evaluated by ANOVA with Tukey’s post-test.

Variations in dietary intake (∆), caloric efficiency, body weight variations (∆), TNF-α in retroperitoneal adipose tissue, as well as the concentration of malondialdehyde in plasma and retroperitoneal adipose tissue, and concentration of sulfhydryls and superoxide dismutase in plasma, showed non-parametric distribution, so the Kruskal–Wallis test with Dunn’s post-test was used to verify the difference between the evaluated groups. Statistically significant differences were considered when *p* ≤ 0.05. Statistical tests were performed using the GraphPad Prism software, version 5.0.

## 5. Conclusions

Based on the above, it is possible to infer that the carotenoid extract of the Cantaloupe Melon (*Cucumis melo* L. var cantalupensis) presented better results due to nanoencapsulation, given that the group of animals that received the EPG containing low CE concentrations, showed negative variation for food consumption, caloric efficiency, and body weight during the evaluated period, promoting improvement in adiposity and consequently in the systemic inflammatory response. In addition, it was observed that there was an improvement in the histopathological parameters of adipose tissue, providing indications of less adipocyte hypertrophy, characterized by the presence of a more prominent focal area of multilocular adipocytes for animals receiving EPG.

Thus, the present study becomes innovative from a technological point of view for EPG, but mainly about its functionality in an experimental in vivo model of chronic inflammation, bringing a new aspect for the future application of this nanoparticle, showing it as a possible nutraceutical with a view to its application in clinical studies involving inflammatory diseases related to the accumulation of adipose tissue.

## Figures and Tables

**Table 1:** Variation of dietary intake, caloric intake, and caloric efficiency of Wistar rats with inflammation induced by HGLI diet; adults (31 weeks) on the first and last day of the experiment, submitted to different treatments after ten days of investigation.

Groups	∆Dietary Intake (g) Mean (SD)	Caloric Intake (kJ/Kcal of Intake) Mean (SD)	Caloric Efficiency (kJ/g of Weight) Mean (SD)
No treatment	3.90 (2.61) ^a^	255.48 (18.22) ^a^	21.97 (62.91) ^a^
Conventional treatment	4.13 (6.73) ^a^	290.70 (64.77) ^a^	1.33 (58.57) ^a^
Test treatment 1 (CE)	0.40 (4.52) ^a^	270.22 (25.32) ^a^	−11.10 (72.80) ^a^
Test treatment 2 (EPG)	−3.00 (3.03) ^a^	239.51 (33.64) ^a^	−38.20 (31.98) ^a^

No treatment: HGLI diet + 1 mL of water by gavage; conventional treatment: nutritionally adequate diet (Labina^®^ feed) + 1 mL of water per gavage; test treatment 1: HGLI diet + 1 mL of CE at a concentration of 12.5 mg/kg by gavage; test treatment 2: HGLI diet + 1 mL of EPG at a concentration of 50 mg/kg by gavage. HGLI diet: mixture composed of Labina^®^, condensed milk, and sugar (1:1:0.21 *w*/*w*/*w*); HGLI: high glycemic index and high glycemic load diet; CE: crude extract rich in carotenoids from Cantaloupe melons; EPG: crude extract rich in carotenoids from Cantaloupe melons nanoencapsulated in porcine gelatin. Values were expressed as mean (standard deviation). Equal letters in the same column indicate no significant difference between the groups evaluated for each parameter (*p* > 0.05). For the evaluation of data normality, the Kolmogorov–Smirnov test was used. Variations in dietary intake (∆) and caloric efficiency showed a non-parametric distribution, so the Kruskal–Wallis test after Dunn’s test was used to verify differences between the evaluated groups. Caloric intake showed a parametric distribution; therefore, the ANOVA test with Tukey’s post-test was used to determine significant differences. Values of *p* ≤ 0.05 were considered statistically significant.

**Table 2:** Variation, loss, and gain of body weight (g) of Wistar rats with inflammation induced by the HGLI diet; adults (31 weeks) on the first and last day of the experiment, submitted to different treatments after ten days of investigation.

Groups	∆Weight (g) Mean (SD)	Average Weight Loss (g)	Average Weight Gain (g)
No treatment	1.40 (9.61) ^a^	8.00 (1.41)	7.70 (6.03)
Conventional treatment	−3.00 (10.46) ^a^	11.00 (8.49)	5.00 (0.00)
Test treatment 1 (CE)	−5.20 (11.82) ^a^	12.30 (9.29)	5.50 (2.12)
Test treatment 2 (EPG)	−11.00 (8.40) ^a^	11.00 (8.40)	-

No treatment: HGLI diet + 1 mL of water by gavage; conventional treatment: nutritionally adequate diet (Labina^®^ feed) + 1 mL of water per gavage; test treatment 1: HGLI diet + 1 mL of CE at a concentration of 12.5 mg/kg by gavage; test treatment 2: HGLI diet + 1 mL of EPG at a concentration of 50 mg/kg by gavage; HGLI diet: mixture composed of Labina^®^, condensed milk and sugar (1:1:0.21 *w*/*w*/*w*); HGLI: high glycemic index and high glycemic load diet; CE: crude extract rich in carotenoids from Cantaloupe melons; EPG: crude extract rich in carotenoids from Cantaloupe melons nanoencapsulated in porcine gelatin. The values obtained for ∆ body weight (final body weight − initial body weight) were expressed as mean (standard deviation). The average weight loss and gain (g) results were expressed as mean (standard deviation) based on the number of animals from the same group that lost or gained weight. Equal letters in the same column indicate no significant difference between the groups evaluated for each parameter (*p* > 0.05). For the evaluation of data normality, the Kolmogorov–Smirnov test was used. Variations in body weight (∆) had a non-parametric distribution, so the Kruskal–Wallis test and Dunn’s post-test were used to verify differences between the evaluated groups. Values of *p* ≤ 0.05 were considered statistically significant.

## Data Availability

Not applicable.

## Supplementary Materials

The supporting information can be downloaded at: https://www.mdpi.com/article/10.3390/ijms241310657/s1.

## Abbreviations

3Rs	Replacement, reduction, and refinement
ABTS	2,2′-azino-bis (3-ethylbenzothiazoline) 6-sulfonic acid
BCA	bicinchoninic acid
BHT	Butylated hydroxytoluene
BSA	Bovine serum albumin
CE	Crude extract rich in carotenoids from Cantaloupe melon
CEUA	Ethics in Animal Use Committee
DPPH	2,2-diphenyl-1-picrylhydrazyl
EDTA	ethylenediaminetetraacetic acid
ELISA	Enzyme-linked immunosorbent assay
EPG	Crude extract rich in carotenoids from Cantaloupe melon nanoencapsulated in porcine gelatin
FA 1	Aqueous phase 1
FA 2	Aqueous phase 2
FTIR	Fourier Transform Infrared Spectrophotometry
HGLI diet	High glycemic index diet and high glycemic load
HRP	Conjugated peroxidase
IC50	The amount necessary to inhibit by 50%
IE	Incorporation efficiency
IKK	IkappaB kinase complex (IKKβ and IKKα)
IL-10	interleukin 10
IL-6	interleukin 6
KCl	potassium chloride
MDA	malondialdehyde
NaCl	sodium chloride
NF-κB	Nuclear factor Kappa B
NutriSBioativoS	Nutrition and Bioactive Substances for Health
O/W	Oil/Water emulsification
PPARα	Peroxisome proliferator-activated receptor alpha
PPARγ	Peroxisome proliferator-activated receptor gamma
RXR	Retinoid X receptor
SDS	sodium dodecyl sulfate
SISGEN	National System of Genetic Heritage Management and Associated Traditional Knowledge
SOD	superoxide dismutase
TBA	thiobarbituric acid
TBARS	thiobarbituric acid reactive substances
TCA	trichloroacetic acid
TNF-α	Tumor Necrosis Factor-alpha
UCP1 and UCP2	Uncoupling proteins 1 and 2
UFRN	Federal University of Rio Grande do Norte
UnP	Potiguar University
UPLC	Ultra Performance Liquid Chromatography
WAT	White adipose tissue
