# Supplementary material for: Efficacy of Carotenoid-Loaded Gelatin Nanoparticles in Reducing Plasma Cytokines and Adipocyte Hypertrophy in Wistar Rats
Source: Int J Mol Sci. 2023 Jun 26;24(13):10657. doi: 10.3390/ijms241310657 (PMC10341683; doi:10.3390/ijms241310657)
Supplement: Supplementary file 1 [file ijms-24-10657-s001.zip › ijms-2290867-supplementary.pdf]

Supplementary Materials

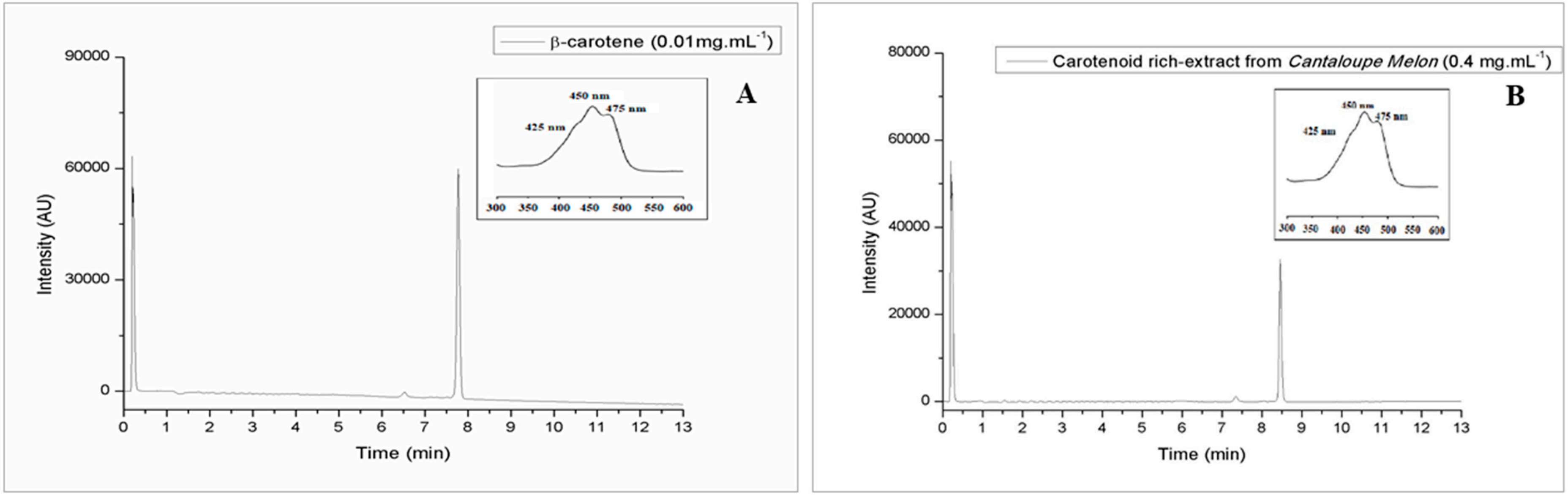

Figure S1. Chromatograms obtained by UPLC. **A**. standard β-carotene (Sigma®); **B**. β-carotene in crude extract rich in carotenoids from Cantaloupe melon pulp. The linear equation obtained from the calibration curve performed with the standard β-carotene was y = 10,000,000x − 77163 (R^2 = 0.998) [13].
